# The new Interventional Radiology/Diagnostic Radiology dual certificate: “higher standards, better education”

**DOI:** 10.1007/s13244-015-0450-9

**Published:** 2016-01-08

**Authors:** Lucy Di Marco, Michael Bret Anderson

**Affiliations:** Centre Hospitalo-Universitaire Dijon France and Visiting research scholar Department of Radiology and Radiological Science, Medical University of South Carolina, Charleston, SC USA; Department of Radiology and Radiological Science, Medical University of South Carolina, Charleston, SC USA

**Keywords:** Medical education, Internship and residency, Radiology, interventional, Certification, IR/DR certificate

## Abstract

**Abstract:**

The American Board of Medical Specialties (ABMS) has approved a “new” residency: dual Certification in Interventional Radiology (IR) / Diagnostic Radiology (DR). This IR/DR program intends to better prepare future IR doctors. Residency programs can apply for the IR/DR program beginning in 2015, with full nationwide implementation of the IR/DR residency likely to ramp up by 2022. The IR/DR dual certificate can be attained via an “Integrated” or “Independent” IR/DR residency pathway. The IR/DR training pathway may lead the way to the future training of IR/DR physicians worldwide. Certainly, the ABMS approval of a “new” residency is rare and worth attention; however, as with all things new, questions and concerns will be raised.

***Main messages*:**

• *The ABMS approved a “new” residency, the IR/DR dual Certification.*

• *This IR/DR program intends to better prepare future IR doctors.*

• *Full nationwide implementation of the IR/DR Residency is likely by 2022.*

• *The IR/DR certificate can be attained* via *the Integrated or the Independent residency pathway.*

• *These programs may lead the way to the future training of IR/DR physicians worldwide.*

## Introduction

Ring and Keran in 1985 predicted that “The development of interventional radiology (IR) services that provide inpatient and outpatient care will establish IR as an important primary service and improve patient care.” [1] IR is steadily on the rise as the discipline of choice to diagnose and treat a vast array of vascular, oncologic and neurologic conditions, performing procedures such as thrombectomy, intra-arterial chemotherapy and embolization, and central venous access. Compared with surgical techniques, IR procedures are less invasive and often safer [2] and are considered among “some of the more *cost- effective* procedures in medicine today” [3], providing high clinical value defined by balancing cost with clinical benefits such as clinical outcomes (lives saved, complication rates, recovery periods, and duration of hospital stays), quality-adjusted life-years, years off dialysis, etc.

Considering the rapid expansion of this specialty and the adoption of its novel techniques into medical guidelines, patients have a right to expect well-trained Interventional Radiologists who will practice safely and effectively with high quality and good outcomes.

IR doctors are not only expected to take care of patients during procedures but also before and after, clinical patient management being an ever more important part of their duty. IR is recognized throughout the world as a distinct subspecialty of radiology, requiring a specific fund of knowledge and procedural skills separate from the diagnostic radiology curriculum. However, not all countries follow a standard curriculum of IR training or perform an assessment by means of examination at the end of IR training. In Europe, for example, though IR achieved subspecialty status and became a subdivision of radiology at the Union of European Medical Specialists in 2009, to date the European Board in Interventional Radiology (EBIR) examination is not required to practice in most European countries. However, as illustrated by the official motto of the European Union « United in diversity », the rules for a license to practice in each country depend on national independent medical authorities. UK trainees, and even trainees from Australia and New Zealand since 2015, for example, are encouraged by their respective Society of Interventional Radiology to take the European Board of Interventional Radiology (EBIR) examination [4–6].

In this paper the authors will outline the recent and ongoing substantial changes in United States (US) Graduate Medical Education (GME), moving toward a more integrated and clinically focused IR/DR training program with dual IR/DR board certification, intended to prepare future IR doctors to manage complex patients and cases that might otherwise not be mastered proficiently in the course of a 1 year-fellowship.

## Evolution and recognition of subspecialty status for interventional radiology in the U.S.

The American Board of Medical Specialties (ABMS) recognized Vascular and Interventional Radiology (VIR) as a subspecialty in 1994.

In 2000, the Society of Interventional Radiology (SIR) created a Voluntary Clinical pathway in IR including 29 months of training in DR and 24 in IR - a modification of the existing Holman pathway. In 2005 a DIRECT (Diagnostic and IR Enhanced Clinical Training) pathway was approved by the American Board of Radiology (ABR) including 27 months in DR and 21 months in IR. Neither pathway was widely adopted [7], and in 2006 the SIR began working on a proposal for a primary certificate in IR, which was subsequently rejected by the ABMS in 2009. The proposal was resubmitted and finally approved as a primary specialty in 2012. In 2013, the ABR announced that it would dual certify IR doctors in IR and DR, reflecting that the essential components of competency address expertise in diagnostic imaging, as well as the full scope of interventional radiology to include the evaluation and clinical management of patients. The Post Graduate Years (PGY) 2–4 are common to DR and IR, with a PGY5 year intended to allow sub-specialization in areas of interest with an entire PGY6 year dedicated to IR.

DR residents are provided the opportunity for optional advanced training in any diagnostic radiology subspecialty area during PGY 5. IR/DR residents complete DR requirements including nuclear medicine and mammography during PGY 5 and begin their advanced training in IR. During PGY 5–6, the IR/DR curriculum includes an Intensive Care Unit (ICU) rotation as well as “IR-related” electives. These electives can be selected from specialties within or outside the radiology department.

Any rotation providing experience in clinical or procedural care that overlaps with or is complementary to interventional radiology can be pursued. At the authors’ institution, rotations in vascular surgery, medical oncology, transplant surgery, luminal gastroenterology, and research focused on IR topics are being considered.

As with diagnostic radiology, residents take the American Board of Radiology core exam at the end of the PGY4 year. Graduates of an IR Residency qualify to take the IR/DR examination offered by the American Board of Radiology (ABR), recognizing competency in both diagnostic radiology and interventional radiology.

The Accreditation Council for GME (ACGME) approved the proposal in 2013 and program requirements for the new IR/DR residency in 2014 [8].

The new IR/DR certificate will now be one of four specialty primary certificates offered by the American Board of Radiology, along with neuroradiology, nuclear radiology, and paediatric radiology. Currently only two ACGME radiology fellowship matches exist, IR and neuroradiology. Over 98 % of IR fellowship positions were filled in 2015, with 270 applicants applying for 234 positions in 82 programs. [9]

## Transition to the IR/DR residency

There will be of necessity a gradual transition from VIR fellowship training to IR/DR residency training. The transition will vary from institution to institution, evolving over time. Programs cannot recruit residents until ACGME accreditation is achieved. Full nationwide implementation of the IR/DR Residency is likely to ramp up to 2022, and all future IR training will ultimately lead to an IR/DR certificate. Accreditation of the historical “one-year fellowships” will be discontinued and the last one-year fellows would graduate in 2020 [10].

The IR/DR certificate can be attained via one of two pathways, the Integrated IR Residency, and the Independent IR Residency; residency programs can apply for either or both of these pathways.

## Integrated IR residency

A candidate would match directly from medical school into an *Integrated* program through the National Resident Match Program (NRMP). After completion of an ACGME-accredited PGY1 internship, the resident would complete a five-year training program, including three years (PGY2 – PGY4) of core DR training followed by two years (PGY5 – PGY6) of training in interventional radiology (IR), including procedural rotations, an ICU rotation, and clinical care experience (both in-patient care and outpatient). Within a single institution, it will be possible to transfer between the IR and DR residencies during the PGY2-PGY4 years.

## Independent IR residency

The two-year *Independent* program (PGY6-PGY7) can be entered at completion of a DR residency. DR residents who meet requirements for the Early Specialization in IR (ESIR) program during their diagnostic training may receive credit for the first year of the two-year independent IR program.

The Independent program requires the same procedural and clinical rotations of the PGY5-PGY6 years of the Integrated program. The independent residency experience may take place at an institution other than where the DR residency was completed.

## IR/DR examination

The IR/DR Certifying Examination will be administered three months post completion of the IR residency and will include the computer-based Essentials of Diagnostic Radiology module and the Noninterpretive Skills module; these same modules are administered to DR candidates on the Certifying Examination.

IR residents will sit for the same Core Examination as their DR peers, typically after completion of the first three years of DR residency.

The IR Certifying Examination is planned to consist of both a computer-based portion and an oral examination, while DR residents no longer take an oral examination. The new IR/DR oral examination is expected to be “longer” than the current version of the oral exam administered to candidates for the Certificate of Added Qualification in VIR, with greater emphasis on clinical patient management.

ABR diplomats presently holding the VIR CAQ will automatically receive the new IR/DR certificate as long as Maintenance of Certification requirements are being met.

## Discussion

Certainly any time the ABMS establishes a new residency training program, some questions and concerns will be raised. Will IR residents lack commitment to DR? For example, will IR residents become exclusively focused on procedural and clinical care and neglect to devote themselves to developing the diagnostic skills necessary of a complete radiologist? Conversely, will medical students be able to commit to IR while in medical school? How will resident transfers within their own or between institutions impact residency programs? The residency programs seeking early IR/DR accreditation will have to address these issues [11]. The authors suggest that these programs will lead the way in the training of the future IR/DR physician, setting the standard in competency and proficiency in diagnosis, intervention, and patient care. Perhaps the European radiology community will be interested in pursuing dual certification in the near future.
